# Genotypic Comparison between *Streptococcus suis* Isolated from Pigs and Humans in Thailand

**DOI:** 10.3390/pathogens9010050

**Published:** 2020-01-09

**Authors:** Anusak Kerdsin, Dan Takeuchi, Aniroot Nuangmek, Yukihiro Akeda, Marcelo Gottschalk, Kazunori Oishi

**Affiliations:** 1 Faculty of Public Health, Kasetsart University Chalermphrakiat Sakon Nakhon Province Campus, Sakon Nakhon 47000, Thailand; 2 Research Institute for Microbial Diseases, Osaka University, Osaka 565-0871, Japan; dantake@biken.osaka-u.ac.jp (D.T.); akeda@biken.osaka-u.ac.jp (Y.A.); 3 Phayao Provincial Livestock Office, Phayao 56000, Thailand; anirootm@yahoo.com; 4 Department of Infection Control and Prevention, Graduate School of Medicine, Osaka University, Osaka 565-0871, Japan; 5 Faculty of Veterinary Medicine, University of Montreal, Montreal, QC H3T 1J4, Canada; marcelo.gottschalk@umontreal.ca; 6 Toyama Institute of Health, Toyama 939-0363, Japan; toyamaeiken1@chic.ocn.ne.jp

**Keywords:** serotype, genotype, *Streptococcus suis*, sequence type (ST), multilocus sequence typing (MLST), pulse-field gel electrophoresis (PFGE)

## Abstract

*Streptococcus suis* is a zoonotic pathogen of economic significance to the swine industry. The number of infected cases is increasing in humans worldwide. In this study, we determined the prevalence and diversity of *S. suis* carriage in slaughterhouse pigs in Phayao province, Thailand, where an outbreak occurred in 2007. The overall *S. suis* carriage rate was 35.2% among slaughterhouse pigs. The prevalence rates of serotypes 2 and 14 (the major serotypes infected in humans) were 6.7% and 2.6%, respectively. In both serotypes, 70.4% of isolates of serotypes 2 and 14 revealed sequence types and pulsotypes identical to human isolates in Thailand. It is suggested that pathogenic strains of *S. suis* are a risk factor for occupational exposure to pigs or the consumption of raw pork products. Food safety, hygiene, and health education should be encouraged to reduce the risk group.

## 1. Introduction

*Streptococcus suis*, an important zoonotic pathogen, causes invasive infections in pigs and in humans in close contact with infected pigs or contaminated pork-derived products [1,2,3]. The number of reported human cases, especially in Southeast Asian countries, has substantially increased in the past few years [1,2,3]. Currently, 29 serotypes of *S. suis* are recognized [4]. Of these, serotype 2 is the most prevalent in *S. suis* human infections, although cases have also been reported caused by serotypes 4, 5, 9, 14, 16, 21, 24, and 31 [1,2,3,5,6,7,8,9].

A previous study reported that slaughterhouse pigs were the main source of *S. suis* serotype 2 human infections in southern Vietnam [10]. A study in Hong Kong also reported an increased bacterial density of *S. suis* in raw pork meats at wet markets where there was a hot and humid climate [11]. Another study reported a high contamination of *S. suis* in pork and edible pig organs in central Thailand [12]. Collectively, the poor quality of food safety controls for raw pork products at slaughterhouses and wet markets in this region is likely providing an important source of this infection. The main route of *S. suis* infection in Thailand has been from the consumption of traditional raw pork or pigs’ blood products [13,14], which are mainly contaminated at the slaughterhouse. A prospective study demonstrated a case-fatality rate of 16.1% and an incidence rate of 6.2 per 100,000 in the general population of northern Thailand [13].

To evaluate the genotypic relationship between *S. suis* isolates recovered from either human or pig origins, *S. suis* isolated from pig tonsils at a slaughterhouses in Phayao province between April 2010 and March 2011 were studied and compared to human isolates recovered in the same region.

## 2. Materials and Methods

### 2.1. Sampling of Pig Tonsils

This study was conducted in Phayao province, northern Thailand between April 2010 and March 2011, within three different periods, as there may be a seasonal change regarding the carriage rate (Table 1). Tonsil samples were collected from all slaughterhouses in the whole province in each period. Pieces of a single tonsil tissue were surgically removed from 580 healthy pigs at 20 slaughterhouses in 9 districts of Phayao province. A single piece of pig tonsil tissue was cut aseptically into small pieces, and subsequently homogenized with Todd-Hewitt broth using a glass tissue homogenizer (Thomas Scientific, Swedesboro, NJ, USA). In total, 0.1 mL of homogenized sample were streaked on the selective medium (NNCC agar) and incubated for 24 to 48 h at 37 °C [15].

### 2.2. Isolation, Identification, and Serotyping of S. suis

Up to 10-alpha-hemolytic colonies from each sample were selected and inoculated on sheep blood agar and further incubated for 24 h. DNA from each colony was extracted using the heat-lysis method [16]. The species of *S. suis* and the corresponding serotypes were determined using multiplex PCR [17]. Where serotypes 2 or 1/2 and 1 or 14 were determined by multiplex PCR, the exact serotype was also confirmed by coagglutination using specific antisera [18].

### 2.3. Genotyping of S. suis

Isolates belonging to *S. suis* serotypes 2 and 14, which are commonly associated with human infection, were further analyzed using multilocus sequence typing (MLST) [19]. MLST alleles and the resulting sequence type (ST) were assigned using the *S. suis* MLST database, which can be accessed at https://pubmlst.org/ssuis/. In addition, pulsed-field gel electrophoresis (PFGE) was performed and the pulsotypes were designated as previously described [5,6,13].

### 2.4. Comparison of S. suis Genotypes

STs and pulsotypes of *S. suis* serotypes 2 and 14 from pigs in this study were compared to the genotypes of human *S. suis* serotypes 2 and 14 isolates already available in our database. The STs and pulsotypes of human *S. suis* serotypes 2 and 14 isolates were performed as previously reported [5,6,13].

## 3. Results

### 3.1. Distribution of S. suis and Its Serotypes in Pig Tonsils

Of the 580 pieces of pig tonsil tissue, 204 (35.2%) were culture-positive for *S. suis* during the three study periods. The proportions of culture-positive were 25.6% for the first period, 41.5% for the second period, and 37.5% for the third period (Table 1). The serotyping results of the 204 *S. suis* isolates are shown in Table 2. Serotypes 1, 1/2, 2, 3, 4, 5, 6, 7, 9, 12, 14, 16, 17, 19, and 24 were found in pig tonsils (Table 2). As shown in Table 1, the overall carriage rate of *S. suis* serotypes 2 and 14 in pig tonsils (which commonly cause human infection) were 6.7% and 2.6%, respectively. Other serotypes previously shown to be able to cause human disease (such as serotypes 4, 5, 9, 16, and 24) were also detected. Of the 204 *S. suis* isolates, serotype 2 (19.1%) was the most common, followed by serotype 7 (15.7%), serotype 9 (14.2%), serotype 16 (9.3%), and serotype 14 (7.3%) (Table 2).

### 3.2. Genotyping of S. suis Isolates from Pig Tonsils

Of the 204 *S. suis* isolates from pigs, we focused on the most important zoonotic *S. suis* isolates, namely serotypes 2 (n = 39) and 14 (n = 15) to further study their genotypic profiles using MLST and PFGE (Table 3). Eight STs (ST1, ST25, ST103, ST28, ST299, ST104, ST105, and ST237) in four clonal complexes (CC), CC1, CC25, CC28, and CC104 were identified within isolates of serotypes 2 and 14. The 39 isolates of serotype 2 were either ST1 (n = 17, 43.6%), ST25 (n = 7, 18%), ST28 (n = 4, 10.2%), ST103 (n = 4, 10.2%), ST104 (n = 5, 12.8%), ST299 (n = 1, 2.6%), or ST300 (n = 1, 2.6%), respectively. Serotype 14 isolates were classified as ST1 (n = 3, 20%), ST105 (n = 8, 53.3%), ST237 (n = 2; 13.3%), ST301 (n = 1, 6.7%), and ST302 (n = 1, 6.7%).

PFGE analysis revealed 27 pulsotypes in these 11 STs. As shown in Table 3, pulsotype A and its series were associated with ST1 and ST237, pulsotypes H and its series were related to ST104, whereas pulsotypes J and its series were commonly found in ST105. On the other hand, ST25, ST28, and ST103 showed diverse pulsotypes whereas ST299, ST300, ST301, and ST302 presented only one pulsotype (Table 3).

### 3.3. Comparison of Genotypes between Pig and Human Isolates

**Figure 1:**
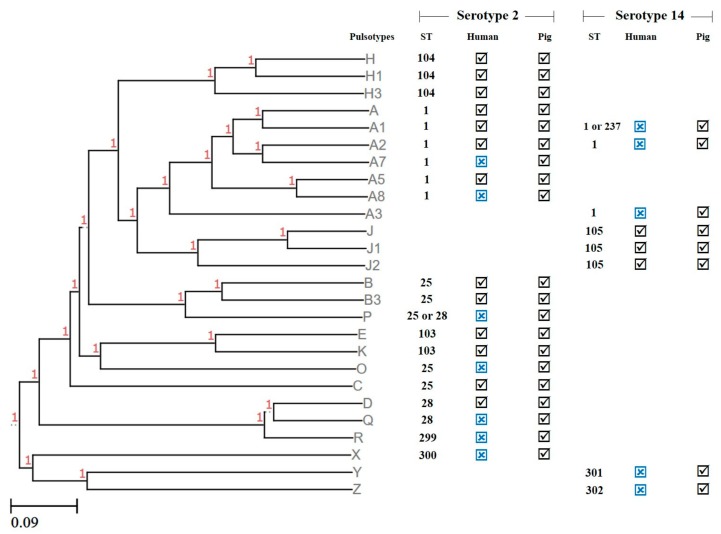
Dendrogram generated from the pulsotypes using the unweighted pair group method with arithmetic mean (UPGMA). Scale bar indicates sequence dissimilarity. 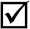 indicates that the genotypes are found in humans or pigs, 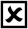 indicates that the genotypes are not found in humans or pigs.

Of the 11 STs found in serotypes 2 and 14, only ST1, ST25, ST28, ST103, ST104, ST105, and ST237 had been previously reported to cause human infections in Thailand. Here, we noticed that, among serotype 2 isolates within STs 1, 25, and 28, only some pulsotypes were associated with human infection. To evaluate whether these STs possessed zoonotic potential, PFGE was used to further characterize these strains and to compare the pulsotypes observed with those of Thai human isolates present in our database and in previous reports (Figure 1) [5,6,13]. As shown in Table 3, 70.4% (38/54) of pig isolates in serotypes 2 and 14 had pulsotypes identical to human isolates. Thirteen (76.5%) out of 17 serotype 2-ST1 isolates, 5 (71.4%) out of 7 isolates of serotype 2-ST25, 1 (25%) out of 4 isolates of serotype 2-ST28, and all strains (100%) of serotype 2-ST103 and ST104 revealed pulsotypes identical to those of human isolates described previously [6].

In the case of serotype 14, ST105 (n = 8) revealed pulsotypes identical to those described for human isolates (Figure 1 and Table 3) [5]. ST237 had a pulsotype (A1) identical to a human strain found in 2009 as shown in our database, as well as in the MLST database (https://pubmlst.org/bigsdb?page=info&db=pubmlst_ssuis_isolates&id=830; Table 3). Interestingly, we found serotype 14-ST1 isolates (n = 3) from pig tonsils; these isolates have never been previously found in humans in Thailand, although they present a pulsotype identical to serotype 2-ST1. Human infections caused by serotype 14-ST1 isolates have been previously described in two meningitis cases in Vietnam and an invasive case in the UK from the MLST database (https://pubmlst.org/bigsdb?db=pubmlst_ssuis_isolates&page=query). The genetic relationship between pig and human isolates with identical STs and pulsotypes in this current study strongly suggests the transmission of *S. suis* serotypes 2 and 14 from pigs to humans (Figure 1).

## 4. Discussion

We determined the prevalence of *S. suis* in slaughterhouse pig tonsils in Phayao province, northern Thailand between April 2010 and March 2011. The carriage rate of *S*. *suis* in healthy slaughterhouse pig tonsils in this study was 35.2% while other studies reported 41% in Vietnam [10], 83% in Canada [20], 13.8% in Korea [21], and 16.9% in China [22]. Differences among studies may be explained, at least in part, by the use of different selective media for bacterial isolation. In this study, we found the carriage rates of *S*. *suis* serotypes 2 and 14 in pig tonsils were 6.7% and 2.6%, respectively. The results showed that among all typable *S. suis*, serotype 2 was the most frequently found, followed by serotypes 7, 9, 16, and 14. Variable results have been obtained in the past in different studies. For example, serotype 2 comprised 8% of isolates in Vietnam [10], 9.1% in Ontario, Canada [20], 3.6% in Korea [21], and 13.7% in China [23]. Serotype 14 carriage in pigs was 3.9% in China [23] and 1% in Canada [24].

Many studies have been carried out in northern Thailand. A previous study reported that serotype 23 (10.2%) was the most prevalent, followed by serotypes 9 (8.2%), 7 (8.2%), and 2 (5.6%) among typable *S. suis* isolates found in healthy pig tonsils in Phayao province [25]. In contrast, serotype 23 was not detected in our study. A difference in the isolation and serotyping methods, or the collection of pig tonsils in the different period, or variation of serotype carriage rates between pig herds may be attributable to the discrepancy in the detection rates of serotype 23 between the previous study and the current study. Two studies were carried out in Chiang Mai, northern Thailand. The first study showed that in samples from the submaxillary gland of pig carcasses, serotype 3 was the most prevalent (17.5%), followed by serotypes 2 (12.5%) and 4 (10%) [26]. In the second study, Padungtod et al. reported that serotypes 2 (43%) and 7 (14.3%) were more prevalent in healthy pigs than diseased pigs [27].

Another report in northeastern Thailand demonstrated that serotypes 7 (12%), 8 (12%), and 2 (2.4%) were the most predominant typable *S. suis* in healthy pigs [28]. On the other hand, a study from central Thailand reported that serotype 16 (11%) was the most frequent serotype recovered from healthy pigs, followed by serotypes 8 (7%), 9 (6%), and 3 (5%) [29]. This suggested that the distribution of *S. suis* serotypes varies in each geographical area (and even within a given area) in Thailand. Supporting this result, the high incidence of Thai patients with *S. suis* serotypes infection may be in concordance with the high prevalence of pathogenic *S. suis* carriage in pigs in the endemic area, especially in northern Thailand.

A previous study of *S. suis* infection in humans in Phayao province from 2010 to 2013 revealed 60 cases infected by serotype 2 out of 71 cases (84.5%) and by serotype 14 in 10 cases (14.1%) [30]. The major STs described were ST1 for serotype 2 and ST105 for serotype 14; however, ST104 was also found as a minor ST in serotype 2 isolates [13,30]. In the present study, we found serotypes and STs in pigs with pulsotypes associated with human infection in Phayao province, especially an important number of serotype 2-ST1 isolates [6,13]. Additionally, the pig tonsils contained *S. suis* serotype 2 ST25, ST28, and ST103, with pulsotypes identical to those described for human isolates in a previous study [6]. This suggests not only a possible transmission of *S. suis* from healthy pigs to humans but also a high prevalence of pathogenic isolates in the slaughterhouse pig population. Therefore, slaughterhouse pigs should be considered as a reservoir of human pathogenic *S. suis* strains. A previous report showed the same genetic profile of MLST, randomly amplified polymorphic DNA (RAPD), and virulence associated gene profile (VAG) from diseased pigs, slaughterhouse pigs, and human cases, suggesting the transmission of *S. suis* isolates from pigs to humans [31]. Therefore, continuous surveillance is important to guide control and prevention measures against infection due to *S. suis* in pigs and humans.

The diversity of serotypes and genotypes of *S. suis* in healthy slaughterhouse pig isolates in our study demonstrated that the population of *S. suis* strains from pigs is more diverse than that of *S. suis* isolated from human patients, suggesting selection in the transmission from pigs to humans [32]. This may be associated with virulence factors, host susceptibility, and environmental tolerance to hot and humid climates. In conclusion, our study confirmed that *S. suis* carriage is common in healthy slaughterhouse pigs. Serotypes 2 and 14, with genotypes identical to human isolates, were also found, suggesting transmission from pigs to humans via the habitual consumption of raw or undercooked pork, and blood and offal products in the form of traditional dishes, or alternatively, via occupational exposure. The prevalence of the pathogen among slaughterhouse pigs indicates that working with these animals is associated with the risk of exposure to this pathogen. Therefore, the following actions should be encouraged: An expanded public health education program, a food safety campaign, and increased hygiene in slaughterhouses and on farms.

## Figures and Tables

**Table 1:** Results of sampling slaughterhouse pig tonsils in Phayao province.

Period	Month/Year	Number of Tonsils, n	Number of Tonsils with *S. suis*, n (%)	Number of Tonsils with *S. suis* Serotype 2, n (%)	Number of Tonsils with *S. suis* Serotype 14, n (%)
I	Apr–Jul, 2010	180	46 (25.6)	13 (7.2)	2 (1.1)
II	Nov 2010–Jan 2011	200	83 (41.5)	16 (8.0)	6 (3.0)
III	Feb–Mar, 2011	200	75 (37.5)	10 (5.0)	7 (3.5)
Total		580	204 (35.2)	39 (6.7)	15 (2.6)

Pig tonsil samples in 3 periods obtained from 9 districts in Phayao province: Dok Khamtai, Muang, Mae Jai, Phukamyao, Chun, Phusang, Pong, Chiang Kham, Chiang Muan.

**Table 2:** Serotype distribution of *Streptococcus suis* isolates from pig tonsils, Thailand.

Serotype	No. of Strains	%
1	3	1.5
1/2	1	0.5
2	39	19.1
3	12	5.9
4	7	3.4
5	2	1
6	4	2
7	32	15.7
9	29	14.2
12	1	0.5
14	15	7.3
16	19	9.3
17	2	1
19	2	1
24	3	1.5
*S. suis*-like	33	16.1

**Table 3:** Distribution of *S. suis* isolates with serotypes 2 and 14 and their genotypes from pig tonsils, in relation to human isolates in Thailand.

Serotype	No. of Isolates (%)	CC	ST	Pulsotypes	Human Isolates	References
2	13 (24.1)	1	1	A, A1, A2, A5	+	[6,13]
	4 (7.4)		1	A7, A8	–	[6,13]
	5 (9.2)	25	25	B, B3, C	+	[6,13]
	2 (3.7)		25	O, P	–	[6,13]
	4 (7.4)		103	E, K	+	[6,13]
	1 (1.9)	28	28	D	+	[6,13]
	3 (5.5)		28	P, Q	–	[6,13]
	1 (1.9)		299	R	–	[6,13]
	5 (9.2)	104	104	H, H1, H3	+	[6,13]
	1 (1.9)	none	300	X	–	[6,13]
Subtotal	39 (72.2)					
14	3 (5.5)	1	1	A1, A2, A3	–	
	8 (14.8)		105	J, J1, J2	+	[5,13]
	2 (3.7)		237	A1	–	
	1 (1.9)	none	301	Y	–	
	1 (1.9)	none	302	Z	–	
Subtotal	15 (27.8)					
Total	54 (100)					

CC = clonal complex; ST = sequence type.
